# Human Infection with Avian Influenza A(H9N2) Virus, Vietnam, April 2024

**DOI:** 10.3201/eid3102.241146

**Published:** 2025-02

**Authors:** Minh Hang Duong, Thi Ngoc Uyen Phan, Trung Hieu Nguyen, Ngoc Hien Nhon Ho, Thu Ngoc Nguyen, Viet Thinh Nguyen, Minh Thang Cao, Chan Quang Luong, Vu Thuong Nguyen, Vu Trung Nguyen

**Affiliations:** Pasteur Institute in Ho Chi Minh City, Ho Chi Minh City, Vietnam

**Keywords:** *Keywords:* influenza, H9N2, avian influenza, whole-genome sequencing, One Health, Vietnam, viruses, respiratory infections, zoonoses

## Abstract

In April 2024, Vietnam confirmed its first human case of influenza A(H9N2) in a 37-year-old man, marking a critical point in regional infectious disease monitoring and response. This case underscores the importance of robust surveillance systems and One Health collaboration in managing emerging zoonotic threats.

Influenza A(H9N2) virus is a low-pathogenicity avian influenza virus endemic in poultry across the world. The virus presents ongoing zoonotic risk; according to the GISAID database (https://www.gisaid.org), ≈100 human cases were detected since 2010, and the virus’s unique evolutionary trajectory shows it could cause pandemics (*1*). The risk for transmission and genetic mixing of avian influenza viruses, including H9N2, among wild birds, swine, and humans highlights the necessity for robust surveillance systems to manage the potential threat of these influenza viruses (*2*–*4*). In Vietnam, H9N2 accounts for 36% of detected avian influenza viruses (*5*) and shares genetic similarities with strains from neighboring countries, particularly China (*3*,*6*). Although human H9N2 cases have been reported in Asia (*7*,*8*), Vietnam had not previously reported a human case until 2024. The first human case of H9N2 influenza in Vietnam was officially confirmed in April 2024, marking a significant event in regional surveillance and response efforts.

A 37-year-old man with a known history of alcohol abuse from Tien Giang Province, Vietnam, experienced fever and cough on March 9, 2024. He sought medical care on March 16 at a provincial hospital, where he received a diagnosis of cirrhosis and was transferred to the Hospital for Tropical Diseases in Ho Chi Minh City, Vietnam, the same day. Initially, his chest radiograph results were unremarkable. However, on March 19, he had pneumonia with dyspnea, fatigue, and extensive alveolar and interstitial damage evident on chest radiograph. He was intubated on March 21; osletamivir treatment was initiated, which improved his condition and enabled him to be weaned from the ventilator. He continued treatment in a negative-pressure intensive care unit; diagnoses were sepsis, influenza A, invasive fungal infection, respiratory failure, pulmonary hemorrhage, gastrointestinal bleeding, acute kidney injury, alcohol-related cirrhosis, and a suspected liver tumor. On May 5, 2024, he was discharged from the hospital to receive palliative care; he died on May 6, 2024.

The patient had not received influenza or COVID-19 vaccinations and had no known exposure to sick or dead poultry. All 15 close contacts, including family, neighbors, a driver, and healthcare workers, remained asymptomatic during 24 days of monitoring; we did not collect samples from them. His neighborhood had informal markets for poultry and other animals, but no avian influenza outbreaks were reported in the preceding 3 months. Testing geese from his home 27 days after symptom onset found no influenza H5 or H9 viruses, whereas 1 sample from nearby poultry markets tested positive for H5N1 virus but none for H9 viruses. 

At the time of this case, southern Vietnam lacked surveillance for severe acute respiratory infection, and influenza-like illness surveillance was limited to 6 sentinel sites, excluding the hospitals involved in this study. The patient was enrolled in Pasteur Institute in Ho Chi Minh City (PIHCM) severe viral pneumonia (SVP) surveillance system, active since 2020, after developing pneumonia with significant respiratory symptoms and chest radiograph findings. On April 1, 2024, PIHCM received the patient’s sample, which had been collected by Hospital for Tropical Diseases on March 21, 2024. On April 8, we performed real-time reverse transcription PCR in accordance with the H9N2 protocol of the Regional Animal Health Office No. 6 Vietnam (RAHO6). The Ct value for H9 was 28.81 and for N2, 29.6, indicating significant viral presence. The sample, cultured on MDCK cells from April 5, underwent whole-genome sequencing from culture passage 2 using MiSeq (https://www.illumina.com) after the US Centers for Disease Control and Prevention pipeline with bioinformatics analysis using the MIRA tool (https://cdcgov.github.io/MIRA) and MEGA version 11 (https://www.megasoftware.net).

We conducted phylogenetic analyses of the viral genes from the sequence we obtained, A/Vietnam/01194/2024 (GISAID accession no. EPI_ISL_19290539), using 37 sequences of H9N2 viruses from the GISAID database that were isolated from humans and avians in Vietnam and Cambodia, as well as from other continents. We assessed the reliability of the phylogenetic trees using 1,000 bootstrap replications. The hemagglutinin gene (accession no. EPI3464272) clustered with lineage viruses from H9N2 human cases A/Cambodia/AHC220057/2022 (accession no. EPI_ISL_17068774) and A/Cambodia/21020301/2021 (accession no. EPI_ISL_1697373) (*8*) (Figure 1). The neuraminidase gene (accession no. EPI3464270) clustered with human viruses A/Hong Kong/VM24002346/2024 (accession no. EPI_ISL_18926219) and A/Environment/Jiangsu/0114/2021 (accession no. EPI_ISL_2650013), chicken viruses from RAHO6 A/chicken/Vietnam/Raho6-18S-0778/2018 (accession no. EPI_ISL_408219) and A/chicken/Vietnam/Raho6-Tg1-V2-S1/2018 (accession no. EPI_ISL_408283) (Figure 2). The genetic distances between our sequence and other human and poultry viruses in Asia and America (Figure 2) suggest household poultry as the likely source of exposure. In addition, our findings highlight the geographic spread of H9N2 virus.

**Figure 1:**
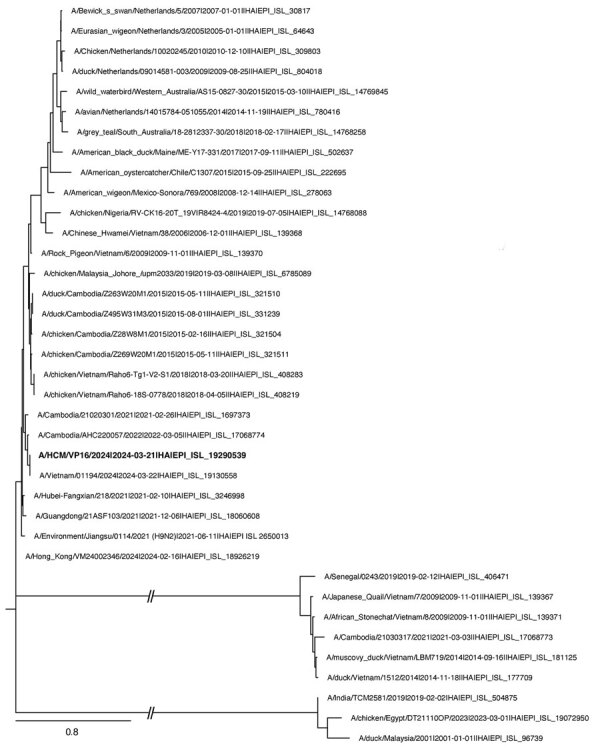
Phylogenetic tree of the hemagglutinin gene of human influenza A(H9N2) virus isolated in Vietnam (bold text) and reference sequences, constructed using the maximum-likelihood method and Tamura-Nei model. The bootstrap consensus tree inferred from 1,000 replicates represents the evolutionary history of the taxa analyzed. Branches corresponding to partitions reproduced in <50% bootstrap replicates are collapsed. We obtained initial trees for the heuristic search automatically by applying neighbor-joining and BioNJ algorithms to a matrix of pairwise distances estimated using the Tamura-Nei model and then selecting the topology with superior log likelihood value. This analysis involved 37 nt sequences; codon positions included were first + second + third + noncoding, for a total of 1,773 positions in the final dataset. Evolutionary analyses were conducted in MEGA 11 (https://www.megasoftware.net). Scale bar represents 0.8 nucleotide substitutions per site.

**Figure 2:**
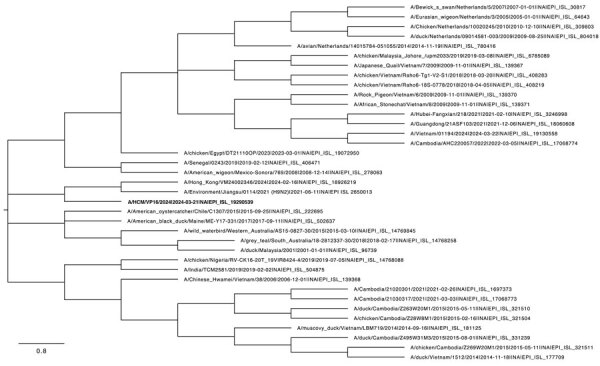
Phylogenetic tree of the neuraminidase gene of human influenza A(H9N2) virus isolated in Vietnam (bold text) and reference sequences, constructed using the maximum-likelihood method and Tamura-Nei model. The bootstrap consensus tree inferred from 1,000 replicates represents the evolutionary history of the taxa analyzed. Branches corresponding to partitions reproduced in <50% bootstrap replicates are collapsed. We obtained initial trees for the heuristic search automatically by applying neighbor-joining and BioNJ algorithms to a matrix of pairwise distances estimated using the Tamura-Nei model and then selecting the topology with superior log likelihood value. This analysis involved 37 nt sequences; codon positions included were first + second + third + noncoding, for a total of 1,524 positions in the final dataset. Evolutionary analyses were conducted in MEGA 11 (https://www.megasoftware.net). Scale bar represents 0.8 nucleotide substitutions per site.

Moving forward, several key research questions arise from this case. First, it is crucial to understand the progression of the disease, particularly the mechanism by which H9N2 influenza contributed to the worsening of the patient’s condition, ultimately leading to multiorgan failure. Second, analyzing samples from close contacts to determine H9N2 positivity is important to identify potential human-to-human transmission, understand transmission dynamics, and guide public health interventions. Although Vietnam lacks a formal One Health rapid response team, this case highlighted the value of cross-sector collaboration. Contributions from public health and animal health sectors, including sequencing expertise and environmental testing, underscore the need to strengthen One Health partnerships for effective surveillance and outbreak response.
